# A Once-Thought Lipoma Turned Malignant Chondroid Syringoma

**DOI:** 10.7759/cureus.37526

**Published:** 2023-04-13

**Authors:** Noelle Provenzano, Emerson T Trimble, Kenneth Zeitzer, Christopher Williamson, Mitchell Goldstein

**Affiliations:** 1 Internal Medicine, Einstein Medical Center Montgomery, East Norriton, USA; 2 Family Medicine, Touro College of Osteopathic Medicine, New York, USA; 3 Radiation Oncology, Einstein Medical Center Montgomery, East Norriton, USA; 4 Orthopedic Surgery, Einstein Medical Center Montgomery, East Norriton, USA; 5 Hematology and Medical Oncology, Einstein Medical Center Montgomery, East Norriton, USA

**Keywords:** risk of malignancy, skin tumor, chondroid syringoma, malignant chondroid syringoma, lipoma

## Abstract

Chondroid syringoma is a relatively rare benign skin appendageal tumor with an incidence of <0.098%. Malignant chondroid syringoma (MCS) arises from cutaneous sweat glands and occurs on the extremities or trunk more commonly in women with only 51 reported cases. Due to the rarity of the disease and lack of published cases of MCS, the diagnostic criteria and treatment protocols are not clear. Based on available recommendations and histological criteria, MCS was diagnosed in a previously classified elbow lipoma following increased size and pain, and skin color changes in a 65-year-old woman.

## Introduction

Chondroid syringoma is a rare benign skin appendageal tumor with an incidence of <0.098% [1]. Its malignant counterpart, malignant chondroid syringoma (MCS), is a tumor that arises from cutaneous sweat glands and occurs on the extremities or trunk more commonly in women, with only 51 reported cases [2].

MCS have high rates of local, regional/nodal, and distant metastasis. MCS on clinical exams present as firm, subcutaneous nodules that are asymptomatic and stable for several years [2]. On histological examination, criteria for chondroid syringoma identification include nests of cuboidal or polygonal cells, tubuloalveolar and ductal structures, a matrix of chondroid or hyaline material, and occasionally keratinous cysts lined with squamous cells [2]. While there are diagnostic markers to aid in the diagnosis of chondroid syringoma, the criteria for MCS are not well established. Histological features suggestive of malignancy including cytologic atypia, infiltrative margins, satellite tumor nodules, tumor necrosis, and involvement of deep structures increase the suspicion of MCS. 

In this case report, we present a 65-year-old female who presented with an elbow mass that was initially diagnosed as a lipoma but later identified to be MCS. Originally evaluated a decade earlier, the nodule on the patient's right elbow became increasingly larger, more painful, and red/purple in appearance, raising suspicion of a malignant process. Following imaging and biopsy, the nodule was reclassified as an MCS, and excision, surveillance for metastatic disease, and radiation therapy were performed. This is the 52nd case of MCS described in the literature. 

This article was previously presented as an abstract at the 2022 ACP Southeastern Regional Posters Day and Doctors Dilemma in Philadelphia, Pennsylvania on October 22, 2022. 

## Case presentation

**Figure 1:**
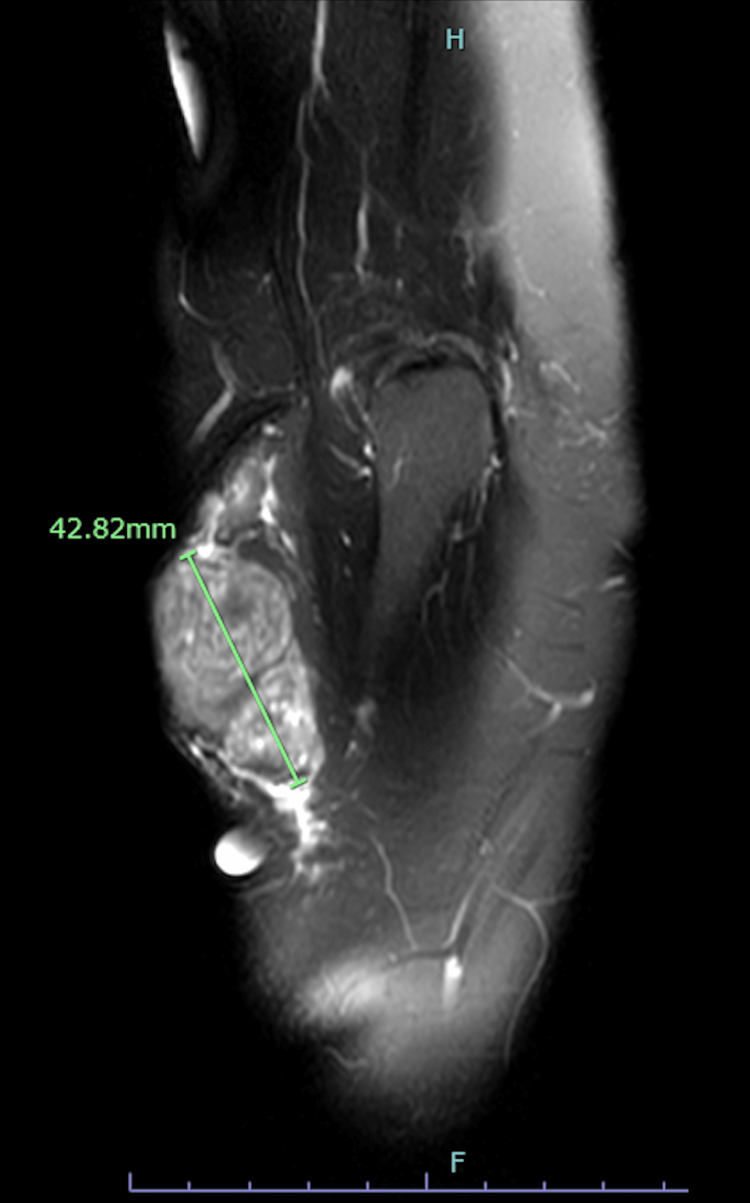
MRI of the right elbow joint revealed a 4.1 x 2.1 x 4.3 cm solid superficial soft tissue mass over the posterior lateral aspect of the proximal forearm

A 65-year-old female with rheumatoid arthritis presented with a painful right elbow mass that had increased in size and pain over the last four months. She described a 10-year history of elbow pain and indicated that the increase in size and pain of the nodule was accompanied by a purple/red color on the overlying skin. Prior to the skin color changes and increasing size, the mass was previously diagnosed as a lipoma 10 years prior. A magnetic resonance imaging (MRI) scan of the right elbow joint revealed a 4.1 x 2.1 x 4.3 cm solid superficial soft tissue mass over the posterior lateral aspect of the proximal forearm (Figure 1).

It also identified several areas of bilobed internal hemorrhage extending to the skin surface, abutting superficial fascia distally. There was no deep compartment extension or intra-articular involvement found on MRI. 

**Figure 2:**
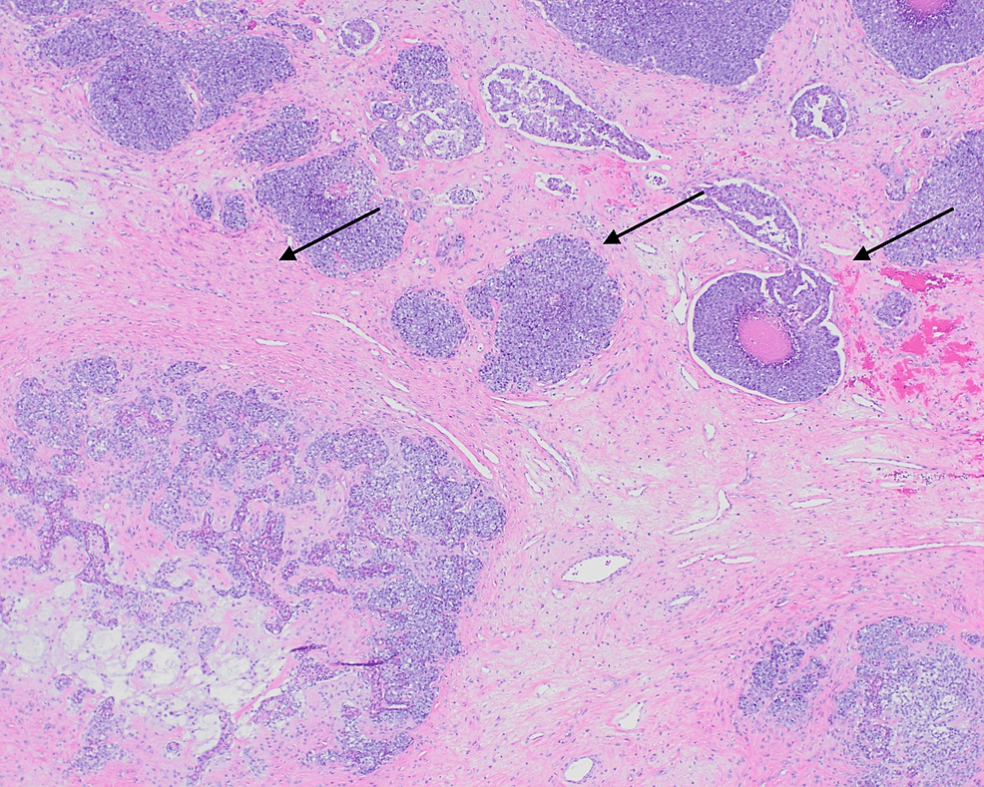
40x H&E stain biopsy demonstrating variable growth patterns

**Figure 3:**
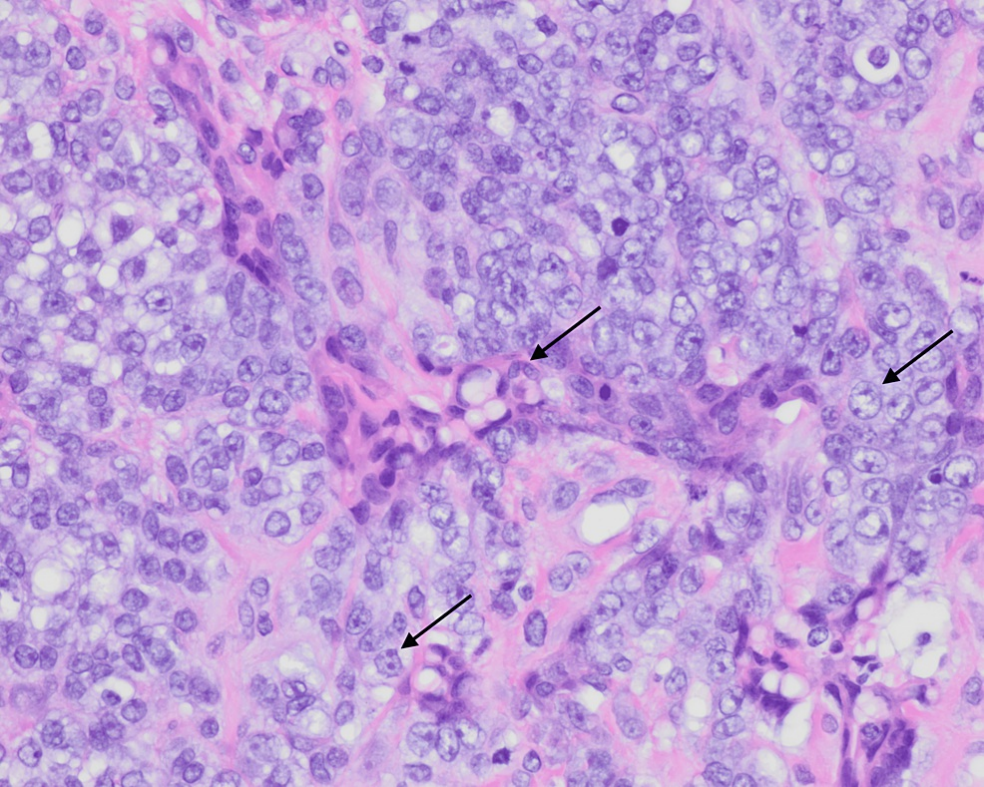
400x H&E stain biopsy demonstrating high mitotic count

Following MRI, the mass was biopsied, revealing a large tumor with several populations of neoplastic cells. Histological analysis of this biopsy identified large aggregates of basaloid cells that show zonal and single-cell necrosis, mitotic activity, and nuclear atypia (Figures 2-3).

**Figure 4:**
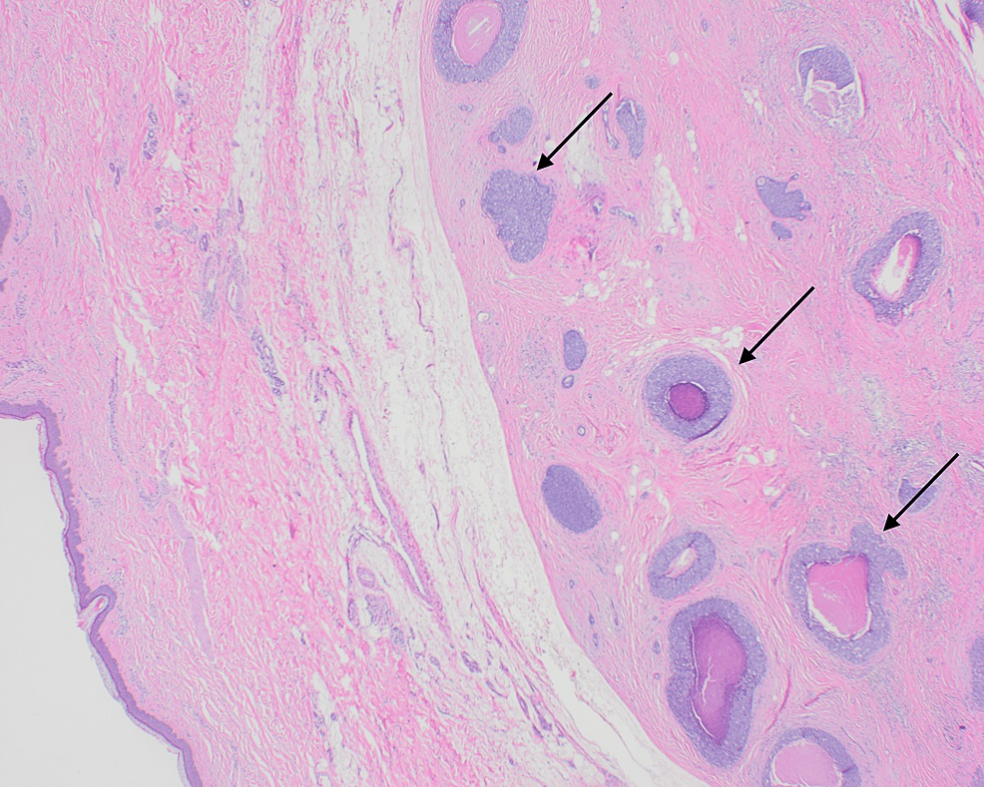
20x H&E stain demonstrating skin, tumor nests, and comedonecrosis

Additionally, other parts of the tumor showed proliferation of epithelial cells that form ductal spaces, along with tumor nests and comedonecrosis (Figure 4).

The biopsy revealed a malignant adnexal tumor that favors the diagnosis of malignant mixed tumor consistent with MCS with multifocal lymphovascular invasion. A CT scan of her chest/abdomen/pelvis was performed post biopsy to screen for metastatic disease and lymphadenopathy, neither of which were identified on CT. 

Re-excision was performed because the initial biopsy involved the surgical margin. Following clear margins, the site was closed and a nuclear medicine body imaging scan was performed. Increased focal uptake was identified in the midshaft of the distal right radius, which was distant from the original site of the tumor, and these findings raised concern for metastasis. However, a subsequent MRI did not identify a mass in the area. Following excision and imaging studies, the patient began treatment with intensity-modulated radiotherapy. 

## Discussion

Infrequently described in the literature, MCS are rare skin tumors that arise from benign lesions and are frequently aggressive. They recur locally in approximately 50% of patients and metastasize in nearly 60% of patients, seeding lymph nodes, lungs, bones, and brain [3]. Despite the rates of metastasis, there are only 51 cases of MCS in literature leaving the exact course of this neoplasm unknown.

While diagnosing the benign counterpart, chondroid syringomas can be aided by employing immunohistochemistry staining for cytokeratins, epithelial membrane antigen (EMA), S‐100, vimentin, carcinoembryonic antigen (CEA), and blood group antigen H, less criteria is available for its malignant counterpart [2] Unlike chondroid syringomas, the diagnostic criteria for MCS is not well established, particularly due to its rarity in practice and publications. Initially, benign tumors can become malignant and metastasize, making the diagnosis of MCS based on histological criteria challenging. However, the presence of histological signs of malignancy increases suspicion of MCS, such as nuclear atypia, cellular pleomorphism, frequent mitotic figures, infiltrative growth, satellite nodules, necrosis, and/or perineural invasion [2]. These features were demonstrated in this patient’s histology thereby aiding the diagnosis. 

The 51 cases of MCS described in the literature provide a basis for common themes of presentation and disease progression. Nuclear atypia and pleomorphism is the most common histological finding in 80% of patients, and MCS are often aggressive tumors, 45% of which recur locally and 52% metastasize without recurring locally [2]. Standard excision practice will likely result in local recurrence (89%), but wide local excision and radiation or amputation can decrease recurrence to 50% and 20%, respectively [2]. In regard to the patient presentations of MCS, the median age of diagnosis is 53 years old with most patients having a median-sized nodule of 4 cm, most likely on the extremities (51%). The median time to treatment is 18 months, and MCS often metastasizes in the first year, primarily to the lymph, lung, bone, and liver [2].

Despite 51 other cases, this is an exceedingly rare tumor with no standardized diagnostic criteria or treatment. Definitive treatment requires surgical removal with wide excision, but adjuvant treatment is not clearly defined. Given the rarity, there is no data for potential benefits of radiation or chemotherapy after surgery. However, radiotherapy has been used both to the primary site, as with other adnexal tumors, and for lymphadenopathy. More data would need to be collected to determine the best course of treatment for this malignancy. 

Although rare, MCS is an important diagnosis to consider in evolving skin masses. 

## Conclusions

This is an exceedingly rare tumor with few case reports in the literature and no standardized diagnostic criteria or treatment. Additional research is needed to determine the best course of treatment for these patients and if routine surveillance, of what are thought to be benign skin masses, is needed. It is an important diagnosis to consider in evolving skin masses. This is the 52nd case of MCS described in the literature. 
